# Hair Analysis to Evaluate Polydrug Use

**DOI:** 10.3390/healthcare9080972

**Published:** 2021-07-31

**Authors:** Giovanna Tassoni, Marta Cippitelli, Gianmario Mietti, Alice Cerioni, Erika Buratti, Emanuele Bury, Mariano Cingolani

**Affiliations:** Forensic Medicine Laboratory, University of Macerata, Don Minzoni 9, 62100 Macerata, Italy; cippitelli.marta@gmail.com (Marta Cippitelli); gianmario.mietti@gmail.com (G.M.); alice.cerioni@libero.it (A.C.); burattierika@gmail.com (E.B.); emanuelebury92@gmail.com (E.B.); mariano.cingolani@unimc.it (M.C.)

**Keywords:** polydrug use, driving people, hair

## Abstract

Polydrug use is a frequent pattern of consumption in Europe. This behavior has mainly been analyzed within restricted groups; more rarely in large populations. Current polydrug use is less studied than simultaneous use. This study focused on the concurrent assumption of polydrug among drivers using hair matrix. Hair matrix, for its biological characteristics, allows to identify illicit drug use more often than other matrices, i.e., urine, and it provides information on the long-term use of them. Hair samples of subjects positive for opiates, cocaine and delta-9-tetrahydrocannabinol (Δ^9^-THC) collected by the forensic toxicology laboratory of the University of Macerata in the period 2010–2020, were analyzed using a gas chromatography-mass spectrometry method. Our results evidenced that a significant part of the examined population (12.15%) used polydrug. A strong predominance of males over females was evident. Polydrug users were more frequently young people. The abuse of two substances was predominant. Cocaine and Δ^9^-THC was the most common combination, followed by cocaine and morphine, and morphine and Δ^9^-THC. The timeframe of polydrug use was also analyzed. Our study shows that polydrug use is a very frequent behavior, and that hair analysis may be a powerful tool to obtain objective biological information of this complex phenomenon.

## 1. Introduction

The use of more than one illicit substance, either concurrently or simultaneously, namely, “polydrug abuse”, is a frequent pattern of consumption in Europe, especially among younger people [1].

The co-use of several substances by an individual over a large period of time could change for different reasons: as a consequence of change in price, availability, legal disposition, the use of separate drugs in different settings or contexts, or reflecting regular multi-substances use associated with drug dependence [1,2]. These factors imply that polydrug use is a complex phenomenon, making it difficult to estimate the real entity and its change over time. Generally, the studies in this field have documented polydrug use among a selected population [3,4,5,6]. There is a paucity of literature of polydrug use in general populations [7,8]. Driving people could effectively represent a general population [9,10,11,12]. Data about the diffusion and characteristics of polydrug use among these subjects may thus contribute to the further knowledge of this phenomenon. According to Italian Code of Road Law art.187, [13] the use of drugs is a valid reason for disqualification from driving, or revocation of the offender’s driver’s license. One of the physical requirements to obtain or re-obtain a suspended driver’s license, according the judgment of a medical commission local Medical Commission, art.119 of the Code of Road Law and DPR 495/92, [14], is the exclusion of illicit drug use by means of toxicological analysis, mainly on urine or hair matrices. While urinalysis informs us of recent or simultaneous exposure to drugs, hair analysis provides information on long term use of illicit drugs, depending on sample length [15]. Hair analysis allows us to identify drug use more often than urinalysis, and may represent a better means by which to control drug abstinence as required by the law [13,14]. Besides the wider diagnostic window, the collection of hair matrix is not invasive, it may be stored at room temperature, it cannot be easily adulterated, and it stores parent drug and metabolites [15]. These proprieties make the hair matrix suitable for the analysis of polydrug use [16,17,18]. Many studies have focused on simultaneous drug use (same time or temporal proximity), the concurrent or sequential patterns of polydrug assumption (30 days or few months) in general population are less reported [7,19]. Furthermore, the entity of concurrent polydrug use on driving behavior has been insufficiently analyzed [1,2]. This paper presents the results of hair analysis of mono and concurrent polydrug use of drivers carried out by the laboratory of the University of Macerata, from people who had had their licenses suspended for driving because resulted positive to illicit drug use over the period 2010–2020. The data represent a considerable population for amount and homogeneity [12]. The database of our laboratory, allows us to analyze the change of the phenomenon over the years, evidencing the new trends and the time change in drug use [12]. Polydrug use is considered to be a particularly highly dangerous risk factor for driving people [20,21], but most studies are related to polydrug use in which one of the substances involved is alcohol [1,2]_._ Less known in the literature is the relevance of concurrent polydrug use on driving behavior when alcohol is not involved [1,20,21].

We have focused on the analysis of the demographic patterns (age, sex), the identification of the most prevalent combinations of polydrug use, its rate of change over time, and the analysis of the risk factors of this behavior. We have focused on data pertaining to opiates, cocaine, cannabis, and their metabolites as required by the protocols of the Medical Commission. They are, in fact, the most abused illicit substances in Italy, while the role of other drugs (i.e., amphetamine, ecstasy) appears less relevant [1,22]_._

## 2. Materials and Methods

### 2.1. Chemicals and Reagents

Nalorphine, proadifen (SKF-525A) (internal standards for opiates and cocaine, respectively), Δ^9^-THC-D_3_ (internal standard for Δ^9^-THC), cocaine, benzoylecgonine, and morphine were purchased from Sigma (Tokyo, Japan). N-methyl, N-trimethylsilyl trifluoroacetamide (MSTFA) and N,O-bis[trime-thylsilyltrifluoroacetamide]w/1%trimethylchlorosilane (BSTFA + 1% TMS) were purchased from Sigma. Methanol, dichloromethane, prop-2-ol, ammonium hydroxide, hexane, ethyl acetate and cyclohexane (purchased from Carlo Erba reagents) were reagent grade. Isolute HCX cartridges (10 mL capacity, 130 mg) were obtained from Thermo (Waltham, MA, USA).

### 2.2. Sample Preparation

All persons registered as permanent residents were given a unique 16-digit identification number. These numbers, along with age, sex, and the substances detected in case of positivity, were entered into a laboratory database containing the results of toxicological analysis of hair samples. All positive cases registered from 2010 to 2020 were used to select those who used more than one substance. The subjects were selected by matching the unique 16-digit number and the number of occurrences in the database. Routinely, we checked a hair sample related to the last 3–4 months (4 cm length). The hair samples were collected from the posterior vertex of individuals being tested for use of drugs, and after washing, they were manually cut into small fragments (50 mg minimum) for drug detection. The samples were incubated overnight in 2 mL of 0.1 N HCl solution at 50 °C, and internal standard (SKF for cocaine, nalorphine for opiates) was added for the detection of cocaine, opiates, and their metabolites. The resulting mixtures were cooled at room temperature and neutralized with 2 mL of phosphate buffer solution, pH 6, and 130 mL of 2 M NaOH (pH 6–7) was added and extracted by means of a solid phase extraction technique. The columns were conditioned sequentially with methanol (2 mL) and phosphate buffer, pH 6 (2 mL). The samples were then slowly drawn through the columns under a low vacuum for at least 2 min. Then, the columns were rinsed sequentially with water (2 mL), 0.1 N HCl (3 mL), and methanol (3 mL). After the columns were completely dried (5 min under full vacuum), the analytes were eluted with 2 mL of a dichloromethane/isopropyl alcohol solution (8:2) with 2% ammonium hydroxide. The eluate was completely evaporated and then derivatized with 50 mL of MSTFA at 60 °C for 20 min. One microliter of the derivatized sample Δ^9^-THC was injected into the gas chromatography/mass spectrometry (GC/MS)apparatus. The residual hair samples used in the previous analyses were used for Δ^9^-THC detection. The samples to which internal standard (Δ^9^-THC-D_3_) was added were subjected to basic hydrolysis (NaOH 1 N solution, at 95 °C for 15 min), cooled at room temperature and subjected to a liquid–liquid extraction method. Three milliliters of an extraction solution of hexane/ethyl acetate (9:1) was added to the samples, which were shaken for at least 15 min. The organic phases were separated. The eluates were completely evaporated and then derivatized with 50 mL of BSTFA + 1% TMS at 60 °C for 20 min. One microliter of the derivatized sample was injected into the GC /MS system.

### 2.3. GC/MS Instrumental and Analytical Conditions

All analyses were carried out by the gas chromatography–mass spectrometry method previously described [12,23]. Drug concentrations in analyzed hair higher than the cutoff values [24,25] were considered to be positive data.

### 2.4. Statistical Methods

Mono- and polydrug user data were expressed as absolute values and proportions. Age data of the two groups were expressed as mean ± standard deviation (SD). Pearson’s chi square test (χ^2^) or the Student’s t test was used to calculate the statistical significance of the demographic factors. Logistic regression (odds ratio (OR), with 95% confidence intervals (CI)) was used to estimate the relationship between the factors analyzed and the likelihood of polydrug use.

## 3. Results

The total number of positive cases was 1432. Of these, 1258 (87.85%) were monodrug users (MDU group). In 12.15% (174) of the cases, more than one substance was found (polydrug users, PDU group). A strong predominance of males over females was evident for both groups (χ^2^ (df = 1) = 4.13, *p* < 0.05. Furthermore, males had a higher likelihood of being polydrug users than females (see Table 1). Logistic regression analysis also showed that females are less likely to become polydrug users than males. The mean ages were 35.00 ± 8.87 years for the MDU group and 33.17 ± 17.26 years for the PDU group. The differences in the mean values of the two groups were statistically significant (t = 2.478, *p* < 0.05), showing that polydrug users were more frequently in the younger ages of the PDU group than in the MDU group. To perform a more in-depth analysis, we divided our age data into different brackets. Age brackets ranged from 18 to 67 years old. The results showed that the 26–35 years age range scored highest, followed by the 36–50 years bracket for both groups. The lowest range was the older age (>51 years) (see Table 1). We compared the young adults (less than 35 years) of MDU and PDU subjects with respect to the respective older subjects (more than 35 years) [1,12,23]. The result shows significant differences. Younger adults in the PDU group were significantly more often polydrug users than older adults (χ^2^ (df = 1) = 7.89, *p* < 0.01), and they showed a moderate risk of using more than one substance (see Table 1).

Cocaine was the most common substance found in all positive MDU cases (60.25%), followed by Δ^9^-THC (26.62%), and morphine (13.12%) (see Table 1). Regarding the PDU group, the abuse of two substances was predominant (98.84%). Cocaine and Δ^9^-THC co-use was the most common (60.25%), followed by cocaine and morphine (33.33%), and morphine and Δ^9^-THC (5.17%). Only 1.16% of cases show the assumption of the three drugs of abuse (see Table 1).

Cocaine was the preeminent drug of abuse in the PDU users (94.83%) compared with cocaine-free cases (see Table 2), and it represent an elevated risk factor for cocaine PDU group compared with cases with cocaine-free PDU group ((5.17%) (χ^2^ (df = 1) = 79.15, *p* < 0.001). The same results were found for morphine users (χ^2^ (df = 1) = 78.75, *p* < 0.001) and Δ^9^-THC users (χ^2^ (df = 1) = 113.57, *p* < 0.001) (see Table 2).

**Figure 1:**
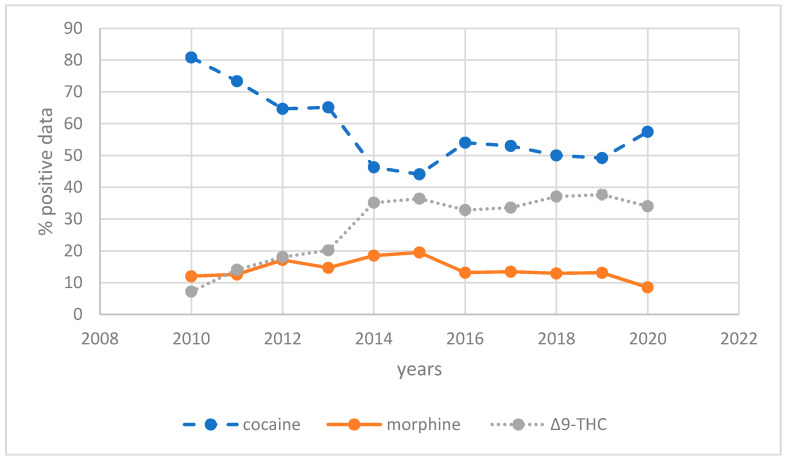
Drugs/year distribution of monodrug users’ group (MDU).

We also analyzed the change in use during the 10-years for the two groups. We have reported the change over the years as a percentage of the single substances positive data with respect to the total number of positive data for each year. The trend of change in the MDU group is shown in Figure 1.

**Figure 2:**
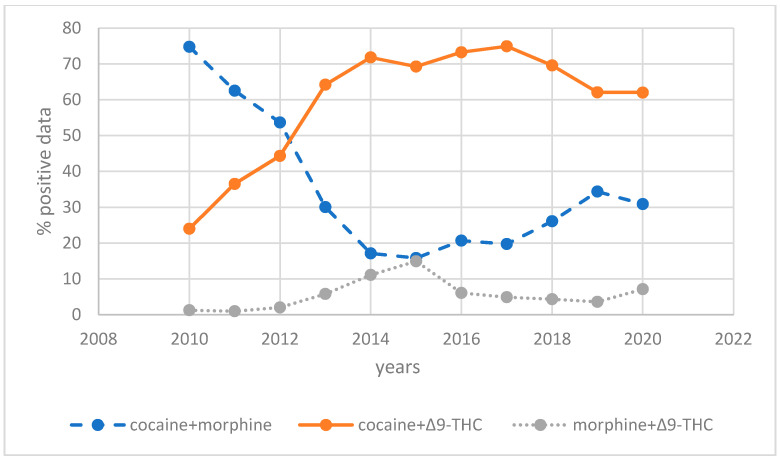
Drugs/year distribution of polydrug users’ group (PDU).

The results show that while the percentages of morphine use were rather stable over the years, cocaine and Δ^9^-THC consumption showed different trends. We found a decreasing trend of cocaine use cases from 2010 to 2014 followed by an increase during the subsequent years. In contrast, Δ^9^-THC data show a constant increasing trend over time. Figure 2 shows the trend of use during the years for the PDU group.

Regarding the PDU group, cocaine and morphine co-use showed a rapid decrease in the number of data points from 2010–2014, and then it remained almost stable for the subsequent 3 years, starting to increase again in 2017. In contrast, we reported a constant progressive increase in the percentage of cocaine and Δ^9^-THC co-use from 2010 to 2014, which remained almost constant at high levels. The data of the co-use of morphine and Δ^9^-THC remained stable at very low levels during all years. The analysis of a 10-years period has allowed us to highlight the progressive increase in cocaine and Δ^9^-THC co-use and the decrease in cocaine and morphine co-use, which has been considered the most frequent drug combination in the PDU group up to 2017. These data reflected the similar decrease in morphine detected alone, an increase in Δ^9^-THC consumption and an almost stable abuse of cocaine of the MDU group.

## 4. Discussion

The aim of this study was to estimate the prevalence of concurrent polydrug use in a large population, the specificity of drug combinations, the evolution of the phenomenon over the time, and the demographic characteristics of the involved populations (age, sex using hair matrix). In particular, the cases were obtained from a database of positive hair samples of driving people found in our lab over a period of 10-years (2010–2020). It is well known that this matrix provides information on the long-term use of drugs of abuse and allows us to identify these drugs more often than urine analysis [15]. The length of hair matrix used in this study (4 cm) allows to detect the presence of illicit drugs taken during the three–four previous months. The results of this study show that 12.5% of the positive cases reported polydrug use. This proportion is higher than those reported in the literature with a similar population [2,20]. The difference could be due to different factors: differences in the substances analyzed, different countries or different time periods analyzed.

The results of our investigation are consistent with other epidemiological data showing that males were significantly more often polydrug users compared with females [8,9], although the evidence is not conclusive in this regard [2]. Moreover, logistic regression analysis also showed that females are less likely to become polydrug users than males. Sex is considered a protective factor underlying substance abuse and addiction [26].

The results of the age bracket distribution are not unexpected. A higher number of polydrug users was found in the age range of 26–35 years old, followed by 18–25 years old. According to the literature, people 18–35 years old are considered young adults [1,20]. The age distribution in our study starts at 18 years as that is the minimum age for a driver’s license in Italy. Our results are consistent with the findings that young adults are more likely to be polydrug users, while older adults are more likely to be monodrug users [1,4,27]. Logistic regression analysis applied to our data confirmed that young subjects in the PDU group (<35 years) were more likely to be polydrug users than older subjects and young MDU subjects. Some investigations have shown that the use of different drugs at different ages depends on the availability of drugs, new trends, drug market supplies, and prices [1,2].

The most common drugs combination found in our study was cocaine and Δ^9^-THC, followed by cocaine and morphine and finally morphine and Δ^9^-THC. The prevalence of cocaine use in association with Δ^9^-THC or morphine was consistent with studies in the literature according to which the assumption of other drugs, especially cannabis, was much higher among cocaine users [20]. Accident risk is higher when cocaine is used in combination with cannabis, with a reinforcement of its detrimental effects [1,5]. More studies have analyzed the effects of the cocaine and heroin co-use [6]. The combination of cocaine and heroin is more neurotoxic than each drug alone [28], with super-addictive or addictive effects [29]. Logistic regression analysis applied to our data confirmed that polydrug use represents a risk factor with respect to monodrug use. Polydrug use has been associated with adverse health outcomes, such as drug dependence [6] and decreased cognitive and motor functioning [30,31]. In particular, the accident risk during driving is higher when cocaine is used in combination with psychoactive substances such as cannabis because the detrimental effects of cocaine can be reinforced [1]. The analysis of single drugs of abuse as risk factors in polydrug behavior shows that all drugs of abuse significantly increase the likelihood of having this behavior, with the major effect due to cocaine. This could be problematic for driving behavior because according to a study, cocaine polydrug users showed a reduced scope of visual attention and compromised ability to control attention compared with free coca polydrug controls [32].

Most of the studies of polydrug use are clinical or involve a particular population (i.e., clinical patients, interviewed students, or adolescents) or are derived from people driving under drugs of abuse involved in crashing, hospital visitors, or arrests. Therefore, they do not allow a picture of polydrug abuse in the general population [10]. The current study, using a more numerous and general population of drivers checked in a random manner, allows a better understanding of the relevance of the complex phenomenon of polydrug abuse and temporal evolution. In particular, regarding this latter aspect, the analysis of a 10-year period has allowed us to highlight the progressive increase in cocaine and Δ^9^-THC co-use and the decrease in cocaine and morphine co-use, which has been considered the most frequent drug combination in polydrug users up to 2016 [33]. These data confirmed a similar decrease in morphine detected alone, an increase in Δ^9^-THC consumption and an almost stable abuse of cocaine.

## 5. Conclusions

The current study showed that hair matrix data of numerous and general population of drivers checked in a random manner, allowed us to analyze the relevance of the complex phenomenon of concurrent polydrug use. For the purpose of our study the hair matrix results are more efficient than other matrices (i.e., urine, blood). Whereas the latter only allow us to detect simultaneous assumption of drug, hair matrix provides a powerful tool to obtain objective biological information on concurrent drug of abuse use. Indeed, its proprieties (ease of its collection, storage at room temperature, the possibility for retrospective monitoring of an accurately determined time period) and the technical characteristics of GC/MS method used in this study (complete resolution of the compound of interest and low time of analysis) allows a more complete identification of drug used. The results of this study showed that the use of more than one substance is higher than those reported in the literature, and seems to be a significant problem in younger males. Cocaine and Δ^9^-THC co-use is the most prevalent, showing a constant increase along the considered period. To the best of our knowledge, the present study is the first that analyzed the concurrent polydrug use in general population. In addition, the results, considering the lengthy time period analyzed and the amount and homogeneity of the data, most likely reflect national conditions of this socially relevant behavior, and may be useful for monitoring mono- and polydrug use of people under periodic control (i.e., work place control). Furthermore, these findings may contribute to the existing literature in concurrent polydrug and on the prevention of this public health problem.

## Figures and Tables

**Table 1:** Characteristics of monodrug users’ group (MDU) and polydrug users’ group (PDU).

Characteristics	MDU (*n* = 1258)		PDU (*n* = 174)		*p*	ODDS-Ratio	
***Variable***	***N***	***%***	***N***	***%***		***OR***	***95% CI***
***Gender***							
Male	1142	90.78	166	95.40	*p* < 0.05	0.477	0.23–0.99
Female	116	9.22	8	4.60			
***Means (years) ± SD***	35.89 ± 8.87		33.17 ± 17.26		*p* < 0.05		
Minimum year	18		20				
Maximum year	67		58				
***Age brackets***							
18–25	214	17.01	40	22.99			
26–35	505	40.14	79	45.40			
36–50	463	36.81	43	24.72			
>51	75	6.04	12	6.89			
18–35	719		119		*p* < 0.001	1.619	2.07–2.27
>36	538		55				
***Substance***							
Cocaine only	758	60.25					
Morphine only	165	13.12					
Δ^9^-THC only	335	26.62					
Cocaine and Δ^9^-THC			105	60.54			
Cocaine and Morphine			58	33.33			
Morphine and Δ^9^-THC			9	5.17			
Morphine and Cocaine and Δ^9^-THC			2	1.16			

**Table 2:** Drug of abuse likelihood risk of monodrug users’ group (MDU) and polydrug users’ group (PDU).

Characteristics	MDU (*n* = 1258)		PDU (*n* = 174)		*p*	ODDS-Ratio	
**Substances risk factor**	***N***	***%***	***N***	***%***		***OR***	***95% CI***
***Cocaine***							
Cocaine	758	60.25	165	94.83	*p* < 0.0001	12.09	19.60–23–87
Cocaine free	500	39.75	9	5.17			
***Morphine***							
Morphine	165	13.12	69	39.65	*p* < 0.0001	4.35	4.41–6–15
Morphine free	1093	86.88	105	60.35			
***Δ^9^-THC***							
Δ^9^-THC	335	26.62	116	66.67	*p* < 0.0001	5.51	2.88–7.73
Δ^9^-THC free	923	73.37	58	33.33			

## Data Availability

No new data were created or analyzed in this study. Data sharing is not applicable to this article.

## References

[1] European Monitoring Centre for Drugs and Drug Addiction (EMCDDA) (2009). Polydrug Use: Pattern and Response. Luxemburg. https://www.emcdda.europa.eu/publications/selected-issues/polydrug-use-patterns-and-responses_en.

[2] Snenghi R., Pelletti G., Frigo A.C., Forza G., Nalesso A., Montisci M., Favretto D. (2018). The dangerous pattern of concurrent use of alcohol and cocaine among drunk-drivers of Northeast Italy. Alcohol Alcohol..

[3] Cicero T.J., Ellis M.S., Kasper Z.A. (2020). Polysubstance use: A broader uderstanding of substance use during opioid crisis. Am. J. Public Health.

[4] Zambon A., Airoldi C., Corrao G., Cibin M., Agostini D., Aliotta F., Movalli M., Biondini F., Bizzi P., Zucchi G. (2017). Prevalence of polysubstance abuse and dual diagnosis in patients admitted to alcohol rehabilitation units for alcohol-related problems in Italy: Changes in 15 years. Alcohol Alcohol..

[5] Lukas S.E., Sholar M., Kouri E., Fukuzako H., Mendelson J.H. (1994). Marihuana smoking increases plasma cocaine levels and subjective reports of euphoria in male volunteer. Pharmacol. Biochem. Behav..

[6] Leri F., Brumeau J., Steward J. (2003). Understanding polydrug use: Review of heroin and cocaine co-use. Addiction.

[7] Karjalainen K., Kuussaaru K., Kataja K., Tigerstedt C., Hakkarainen P. (2017). Measuring polydrug use in general population: A critical assessment. Eur. Addict Res..

[8] Reyes J.C., Peree C.M., Colon M.M., Dowell M.H., Cunsielle F. (2013). Prevalence and patterns of polydrug use in Latin America: Analysis of population-based surveys in six countries. Rev. Eur. Stud..

[9] Karjalainen K.K., Lintonen T.P., Impinen A.O., Lillsunde P.M., Ostano A.I. (2010). Polydrug findings in drugged driving cases during 1977–2007. J. Subst. Use.

[10] Scherer M., Voas P.B., Holden D.F. (2013). Marijuana as a predictor of concurrent substance use among motor vehicle operators. J. Psychoact. Drugs.

[11] Vallancourt L., Viel E., Dombrovski C., Desharmais B., Mireault P. (2021). Drug and driving prior to cannabis legislation: A 5 years review from DECP (DRE) cases in the province of Quebec, Canada. Accid. Anal. Prev..

[12] Tassoni G., Mirtella D., Zampi M., Ferrante L., Cippitelli M., Cognigni E., Froldi R., Cingolani M. (2014). Hair sample in order to evaluate drug abuse in driver’s regranting procedures. Forensic Sci. Int..

[13] D.Leg.285/82. Gazz. Uff. Repubblica Italiana, Suppl. Ordinario n. 114, 18/5/92. https://gazzetta.ufficiale.it.

[14] D.P.R. 495/92. Gazz. Uff. Repubblica Italiana, Suppl. Ordinario n. 302, 28/12/92. https://gazzetta.ufficiale.it.

[15] Kintz P. (2017). Hair analysis in forensic toxicology: Un update review with special focus on pitfalls. Curr. Pharm. Res..

[16] Rust K.Y., Baumgartner M.R., Dally A.M., Kraemer K. (2012). Prevalence of new psychoactive substances: A retrospective study in hair. Drug Test. Anal..

[17] Lendoiro E., De Castro A., Jimenez-Morigosa C., Gomez-Fraguela X.A., Lopez-Rivadulla M., Cruz A. (2018). Usefulness of hair and psychological tests for identification of alcohol and drugs of abuse consumption in driving license regranting. Forensic Sci. Int..

[18] Gili A., Bacci M., Aron K., Nicoletta A., Gambelunghe A., Mercuri I., Gambelunghe C. (2021). Changes in drug use pattern during Covid-19 pandemic in Italy: Monitoring a vulnerable group by hair analysis. Int. J. Environ. Res..

[19] Crummy E.A., Neal T.J.O., Baskin B.M., Ferguson S.M. (2021). One is not enough: Understanding and modeling polysubstance use. Front. Neurosci..

[20] European Monitoring Centre for Drugs and Drug Addiction (EMCDDA) (2014). Drug Use, Impaired Driving and Traffic Accidents. http://www.emcdda.europa.eu/system/files/publications/849/TDXD14016ENN_474631.pdf.

[21] Schultze H., Schumacher M., Urmeew R., Auerbach K., Alvarez J., Bernhoft I.M., de Gier H.D.G., Hagenzieker M., Houwing S., Knoche A. (2012). Driving under the Influence of Drugs, Alcohol and Medicine in Europe-Findings from the DRUID Project. www.emcdda.europa.eu/publications.

[22] Relazione Annuale al Parlamento 2020. www.politicheantidroga.gov.it.

[23] Tassoni G., Cippitelli M., Mirtella D., Froldi R., Ottaviani G., Zampi M., Cingolani M. (2016). Driving under the effect of drugs: Hair analysis in order to evaluate recidivism. Forensic Sci. Int..

[24] Cooper G.A., Kronstandt K., Kintz P. (2012). Society of hair testing guidelines for drug testing in hair. Forensic Sci. Int..

[25] DPR 309/90 art 125. Gazzetta Ufficiale Repubblica Italiana. Supplemento ordinario n.256 del 31/10/1990. Accordo Stato-regioni del 18/09/2008. Allegato A. https://gazzetta.ufficiale.it.

[26] Cotto J.H., Davis E., Dowling G.J., Elcano J.C., Staton A.B., Weiss S.R.B. (2010). Gender effects on drug use, abuse and dependence: A special analysis of results from the national survey on drug and health. Gend. Med..

[27] Kedia S., Sell M.A., Releyea G. (2007). Mono versus polydrug abuse patterns among publicity funded clients. Subst. Abuse Treat Prev. Policy.

[28] Cunha-Oliveira T., Rego A.C., Garrido J., Borges F., Macedoc T., Oliveira C.R. (2010). Neurotoxicity of heroin–cocaine combinations in rat cortical neurons. Toxicology.

[29] Negus S.S. (2005). Interaction between the reinforcing effects of cocaine and heroin in a drug-vs-food choice procedure in rhesus monkeys: A dose-addiction analysis. Psychopharmacology.

[30] Unterrainer M.F., Heibler-Ragger M., Koschutnig K., Fuchshuber J., Ragger K., Perchtold C.M., Papousek I., Weiss E.M., Fink A. (2019). Brain structure alterations in polydrug use: Reduced cortical thickness and white matter impairment in region associate with affective, cognitive and motor functions. Front. Psychiatry.

[31] Preti E., Prunas A., Ravera F., Madeddu F. (2011). Polydrug abuse and personality disorders in a sample of substances-abusing inpatients. Ment. Health Subst. Use.

[32] Colzato S., van den Wildenberg W.P.M., Hommel B. (2009). Reduced Attentional Scope in Cocaine Polydrug Users. PLoS ONE.

[33] Gomez-Talagon T., Fierro I., Gonzalez-Luque J.C., Colas M., Lopez-Rivadulla M., Alvarez F.J. (2012). Prevalence of psychoactive substances, alcohol, illicit drugs, and medicines, in Spanish drivers: A roadside study. Forensic Sci. Int..

